# Systematic Application of DNA Fiber-FISH Technique in Cotton

**DOI:** 10.1371/journal.pone.0075674

**Published:** 2013-09-27

**Authors:** Kai Wang, Wenpan Zhang, Yanqin Jiang, Tianzhen Zhang

**Affiliations:** 1 National Key Laboratory of Crop Genetics and Germplasm Enhancement, Cotton Research Institute, Nanjing Agricultural University, Nanjing, China; 2 Institute of Botany, Jiangsu Province & Chinese Academy of Sciences, Nanjing, China; East Carolina University, United States of America

## Abstract

Fluorescence in situ hybridization on extended DNA (fiber-FISH) is a powerful tool in high-resolution physical mapping. To introduce this technique into cotton, we developed the technique and tested it by deliberately mapping of telomere and 5S rDNA. Results showed that telomere-length ranged from 0.80 kb to 37.86 kb in three species, *G. hirsutum*, *G. herbaceum* and *G. arboreum*. However, most of the telomeres (>91.0%) were below 10 kb. The length of 5S rDNA was revealed as 964 kb in *G. herbaceum* whereas, in *G. arboreum*, it was approximately three times longer (3.1 Mb). A fiber-FISH based immunofluorescence method was also described to assay the DNA methylation. Using this technique, we revealed that both telomere and 5S rDNA were methylated at different levels. In addition, we developed a BAC molecule-based fiber-FISH technique. Using this technique, we can precisely map BAC clones on each other and evaluated the size and location of overlapped regions. The development and application of fiber-FISH technique will facilitate high-resolution physical mapping and further directed sequencing projects for cotton.

## Introduction

Fluorescence in situ hybridization on extended DNA (fiber-FISH) is a powerful high-resolution physical mapping approach. Because extended DNA is the target in fiber-FISH, the small size of chromosome which is difficult to handle in classical cytogenetic analysis is no longer an obstacle to cytological study [1], [2]. In addition, the DNA fiber can be extends approximately 2.5–3.5 kb/µm on slides [1]–[4]. Thus, the resolution of fiber-FISH can reach up to a few of kilo bases [5]. Fiber-FISH has been widely applied in various ways in genome research in animals and plants, including analysis of structure and organization of repetitive sequence, mapping of BAC and chloroplast, analysis of transgenic DNA, measurement of the gap in physical maps (see review in [5]). So far fiber-FISH may be an irreplaceable technique to accurately visualize the copy number of tandem repeats which will be difficult to be figured out by quantitative PCR, southern blot and modern sequencing technique [6]–[9].

Combined with the technique of immunofluorescence assay, fiber-FISH can be used to analyze the epigenetic marks, such as DNA methylation and histone modification [10]–[12]. This technique has been used to visualize the DNA methylation in mouse pericentromeric regions [11] and to examine the relationship between CENP-A and H3-containing nucleosomes in flies and humans [12]. Recently, it was introduced into plant to precisely map the cytosine methylation associated with different centromeric repetitive sequence in maize [10]. The high resolution allowed researcher to observe the effects of different level of cytosine methylation on function centromeric chromatin [10].

Cotton is not only a leading fiber crop worldwide but excellent model plant for genome structure study. Extensive efforts have been devoted on the exploration of cotton genome composition and alteration, including recent global assays of its genomic complexity [13], [14]. FISH as a powerful physical mapping approach has been applied in cotton genomic research, such as chromosome identification [15], [16], distribution of repetitive DNA [17]–[19], and physical map construction [20]. However, all of them were based on metaphase or pachytene chromosomes, which provided a limited resolution [21]. Here, we successfully introduced the high-resolution technique, fiber-FISH, into cotton by conducting a systematic applications, including technique calibration, and physical mapping of telomere and 5S rDNA. We also combined immunofluorescence technique to check the status of cytosine methylation on cotton fibers. In addition, a fast and easy method of BAC molecule fiber-FISH was developed for facilitating assembly of BAC clones and further physical mapping.

## Results and Discussion

### Calibration of the extension degree of cotton fiber

**Figure 1:**
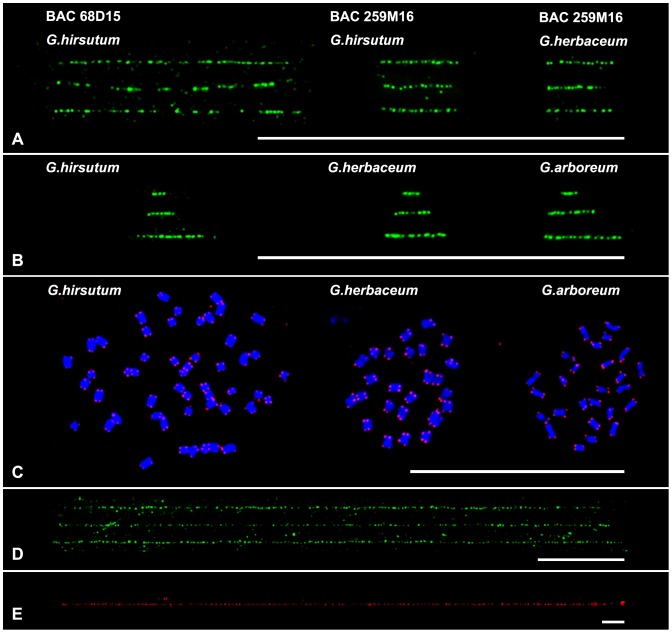
FISH mapping on chromosomes and extended DNA fibers in cotton. A) fiber-FISH signals derived from two BACs 68D15 and 259M16 in *G. hirsutum* and *G. herbaceum*. Three fibers were presented for per hybridization. B) fiber-FISH signals derived from the telomere in three species, *G. hirsutum*, *G. herbaceum* and *G. arboreum*. Three images with different size were presented for per species. C) FISH analysis of telomere (red signals) on metaphase chromosomes of three species, *G. hirsutum*, *G. herbaceum* and *G. arboreum*. No detectable telomere signals were found from internal regions. D) Three fiber-FISH images of 5S rDNA from *G. herbaceum*. E) The longest fiber-FISH image of 5S rDNA from *G. arboreum*. All bars are 50 µm.

Quantitation of physical distance in base pair is an important application for Fiber-FISH. To ensure accurate quantitation of the physical distance, the conversion value of microscopic size (µm) and physical size (kb) have to be established previously using this technique. Two *G. hirsutum* BAC clones 68D15 and 259M16 were used to calibrate the extension degree of cotton fiber. The inserts of clones 68D15 and 259M16 were 95.5 kb and 35.9 kb, respectively, as revealed by sequencing analysis. The average lengths of signals were 29. 7±1.9 µm (n = 25) for BAC 68D15 and 11.0±1.6 µm (n = 25) for BAC 259M16 in fiber-FISH (Figure 1A, Table 1). So the average resolution from these two BAC data was 3.24 kb/µm (Table 1).

**Table 1:** Evaluation of the resolution of extended fiber and molecule by two BAC clones.

BAC Clones	Insert size (kb)	Extended chromatin fiber			linear BAC molecule		
		Average size (µm)	Resolution (kb/µm)	n	Average size^a^ (µm)	Resolution (kb/µm)	n
68D15	95.5	29. 7±1.9	3.21	25	17.7±2.5	5.81	20
259M16	35.9	11.0±1.6	3.26	25	7.7±1.2	5.67	20

a the 7.5-kb vector was included.

The degree of DNA stretching has been measured in plants and animals, ranging from 2.87 to 3.3 kb/µm [1], [2], [4], [22]–[24]. In plants, this technique has been calibrated only in *Arabidopsis* [1], [2] and rice [4]. However, large variation was reported by different researchers in *Arabidopsis*, 3.27 kb/µm [2] and 2.87 kb/µm [1]. The variation was supposed to be due to the short DNA used in analyzing, which generated only a few consecutive fluorescence spots [1], [4]. To minimize the variation, ∼1 Mb contig that contains seven BAC clones was used to calibrate this technique in rice, and result showed it as 3.21 kb/µm [4]. Studies have showed that there was a similar stretching level in several plants, such as *Arabidopsis*, maize, potato, soybean and rice [2], [6]–[8], [25]. In our study, the stretching degree is similar to that obtained in rice, indicating that there was a uniform stretching level in plants.

Because clone 259M16 was derived from the A-subgenome chromosome (12A) [20], we then tried to map BACs 259M16 in two A-genome species, *G. arboreum and G. herbaceum*, which were generally regarded as the exemplars of the A-subgenome progenitors [26], [27]. However, FISH signals only from *G. herbaceum* were detected on both metaphase chromosome (figure not shown) and extended DNA. Interestingly, the length of signal from *G. herbaceum* was 10.3±1.3 µm (n = 20), which is no significant difference (*t*-test, *p* = 0.14) with it obtained in *G. hirsutum*. In addition, the uniform bright signals suggested that the sequence of this 35.9 kb region was highly identical in *G. hirsutum* and *G. herbaceum*.

### Evaluation of cotton telomere length by fiber-FISH

**Figure 2:**
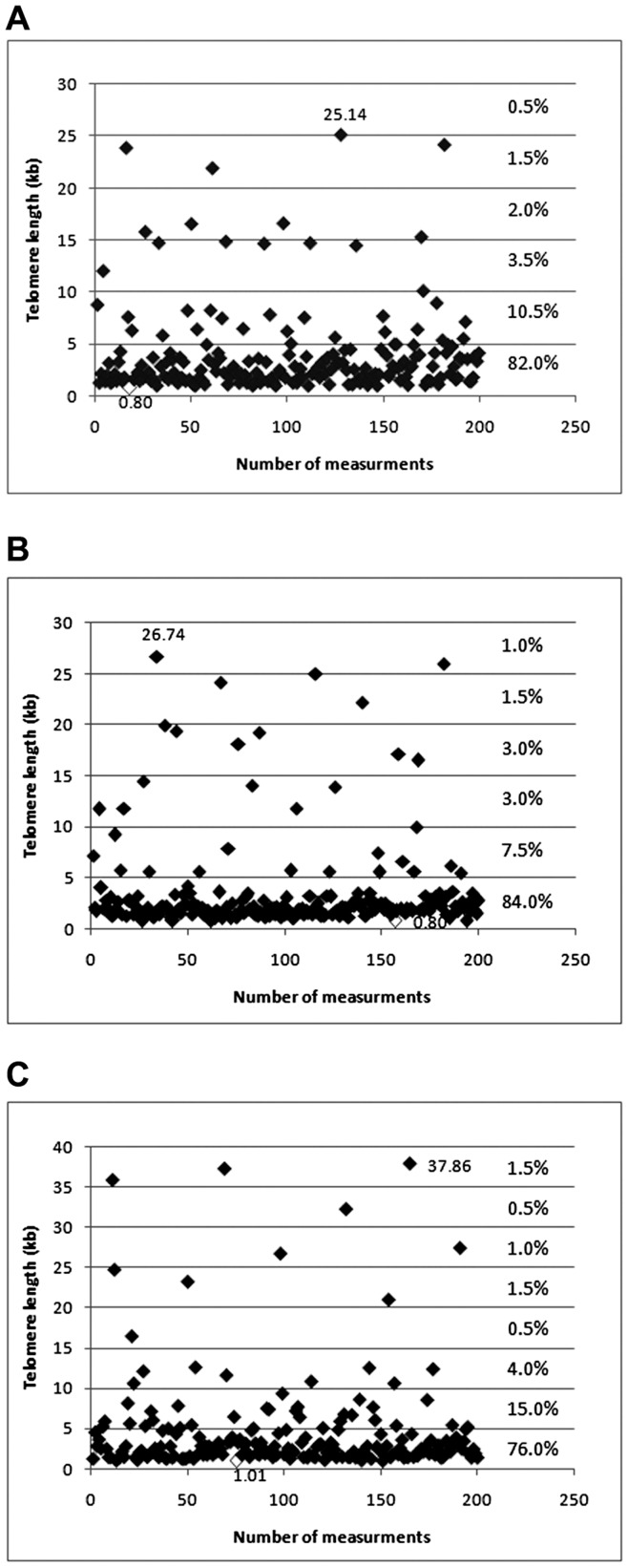
Telomere-size distributions in *G. hirsutum*, *G. herbaceum* and *G. arboreum*. Two hundred telomere signals for *G. hirsutum* (A), *G. herbaceum* (B) and *G. arboreum* (C) were measured from three individual fiber-FISH slides. The percentage of telomere within different size-rang were indicated. The sizes of the longest and shortest telomeres were showed on the corresponding measurements.

*Arabidopsis* telomere which is composed of telomeric repeat arrays of TTTAGGG has been found in most plants [28]. The length of telomere varies dramatically among species, from 2–4 kb in *Arabidopsis* up to 60–160 kb in tobacco [29]. To evaluate telomere length in cotton, an *Arabidopsis*-telomere probe was developed and used in fiber-FISH to check the telomere length of three species, *G. hirsutum*, *G. herbaceum* and *G. arboreum*. Based on 200 measurements, telomere size was revealed as 0.80–25.14 kb in *G. hirsutum*, 0.80–26.74 kb in *G. herbaceum* and 1.01–37.86 kb in *G. arboreum* (Figure 1 and 2). As shown in Figure 1B, all multiple-dot signals showed continuous dotted tracks with uniform florescence intensity, suggesting that the TTTAGGG repeat unit were tandemly reiterated without interruption by other sequences. Therefore, we could deduce that there were 114–5,408 copies of TTTAGGG repeat in these cottons. Overall, all these three species showed similar telomere-size distributions (Figure 2), in which most of the telomere size (92.5%, 91.5.0% and 91.0% in *G. hirsutum*, *G. herbaceum* and *G. arboreum*, respectively) were below 10 kb. However, the average telomere size of *G. arboreum* (4.63 kb) was longer than both of *G. hirsutum* and *G. herbaceum* (3.96 kb and 3.77 kb, respectively). In addition, we found four telomere measurements of *G. arboreum* were >30 kb (Figure 2C), suggesting that there was (were) one or several telomeres with a size >30 kb, whereas no telomeres were more than 30 kb in size in *G. hirsutum* and *G. herbaceum*.

However, a question raised here is how to distinguish the internal telomeric repeats (ITRs) which might exist in cotton genome as it is the case in *Arabidopsis* [30]. To test the existing of ITR, we hybridized the telomere probe onto metaphase chromosomes in FISH. Results showed that no detectable signals were found from internal regions, suggesting no significant fractions of ITRs in *G. hirsutum*, *G. arboreum* and *G. herbaceum* (Figure 1C). Additional the uniform signal intensity from our fiber-FISH also exclude its existing because most of ITRs contained degenerated TTTAGGG arrays [30], which would cause nonuniform signal intensity in FISH.

In plants and animals, a wide range of methods have been developed to measure telomere size such as terminal restriction fragment (TRF), flow-FISH, quantitative FISH and qPCR assay [31], [32]. All the methods, except TRF, have the disadvantage of generating a relative measure of telomere size. The advantage of fiber-FISH is that, unlike RTF assay individual telomere could be measured. In rice, combing with subtelomere-specific sequence, seven telomeres have been characterized by fiber-FISH analysis [33]. The next target for us will be to isolate chromosome-specific subtelomeric sequence, and then to characterize individual telomeres in cotton. The data obtained here will facilitate our further study and also will facilitate further sequencing of the individual telomere regions in cotton.

### Physical length of 5S rDNA in *G. herbaceum* and *G. arboreum*

For 5S rDNA, diploid cotton *G. herbaceum* and *G. arboreum* were analyzed because they have only one 5S rDNA site [16], [17]. Continuous signals were obtained from both species (Figure 1D and 1E), confirming that they are organized as long tandem arrays. For *G. herbaceum*, ten good-quality fibers were measured. Results showed that it spanned 297.5±16.3 µm (n = 10), representing an average of 964.0±52.7 kb (Figure 1D). For *G. arboreum*, however, dramatically long fibers were obtained (Figure 1E). We collected five good-quality fibers, which showed a length from 900.8 µm to 1,035.6 µm (Table S1). The average length was 947.3±52.5 µm, suggesting that 5S rDNA was >3.1 Mb in *G. arboreum*. 5S RNA genes are highly conserved in the plant kingdom, both with respect to length and nucleotide sequence [34], [35]. Sequencing analyzing has shown that the 5S RNA genes of *G. herbaceum* and *G. arboreum* had also a high level of sequence identity (>92%, 297/298 bp) [36]. It means, therefore, that there were ∼3,234 copies of 5S array (298 bp) [36] in *G. herbaceum*. However, in *G. arboreum*, it was at least 3.1-fold longer (>10,300 copies) than that observed in *G. herbaceum*.

In plants, the size estimates of long tandem repeat such as rDNA have mostly been established by means of southern hybridization/southern blot [32]. However, this procedure often overestimated the size due to the positions of restriction sites for rare-cutting enzymes close to but not at the proximal border of repeats. Additionally, for multiple loci, it only provides an overall size estimate, which is not suited for analyzing the length variation at specific chromosomes. These limitations can be overcome by the application of FISH on extended DNA fibers, which provides a more accurate tool to study the molecular organization of target DNA, including tandem repeat with a size below 1 Mb. Combing with a chromosome-specific DNA adjacent with the repeat, fiber-FISH can provide supplementary information on the mapping of repeats to specific chromosomes [33], [37], [38].

One of our goals was to determine the limits of fiber-FISH for measuring the longest DNA in cotton. Previously, DNA molecules >1,000 kb was supposed to be broken during DNA fiber preparation [1], [7]. Our data from *G. herbaceum* showed a length of ∼3 Mb with a relative low standard-deviation (±170.1 kb), indicating that size data was reliable at ∼3 Mb in our experiments.

### Epigenetic status of 5S rDNA and telomere in cotton

**Figure 3:**
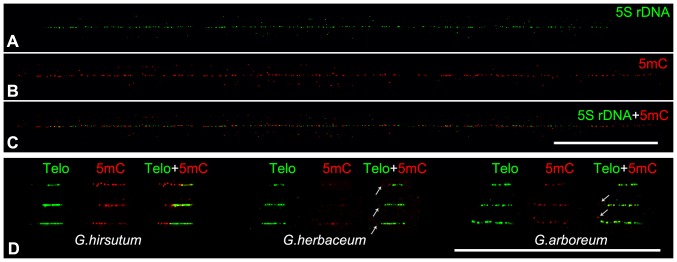
DNA methylation assay of cotton telomere and 5S rDNA. A-C) DNA hypermethylated along the 5S rDNA in *G. herbaceum* revealed by immunodetection of 5 mC on DNA fibers. D) Mapping of DNA methylation in the telomere of three species, *G. hirsutum*, *G. herbaceum* and *G. arboreum*. Three images with different size were presented for per species. In *G. hirsutum*, the adjacent subtelomeric regions were also heavily methylated, whereas only few week dots (arrows) were found in the subtelomeric regions in both *G. herbaceum* and *G. arboreum*. Bars are 50 µm.

Cytosine methylation is a major epigenetic modification of DNA that plays a crucial role in the transcriptional regulation of specific genes, such as inactivation of transposon proliferation, control of genomic imprinting and regulation of gene expression [39]. Although genome-wide analysis of DNA methylation at a single-base resolution has been accomplished in plants [40]–[42], the mapping of cytosine methylation on repeat, especially on long range of tandem repeat DNA still is a challenge. Recently, a DNA fiber-based technique was developed to assess the level of cytosine methylation associated with different centromeric repetitive DNA [10]. Here this technique was introduced in cotton to analyze the cytosine methylation of telomere and 5S rDNA. FISH analysis has revealed that the only 5S rDNA of *G. arboreum* and *G. herbaceum* located in pericentromeric heterochromatin region [15], [36], where abounds in methylated heterochromatin. To evaluate the methylation status, 5S rDNA from *G. herbaceum* was assayed because it has a relative short size, which will ensure us to collect data on the entire 5S arrays. As showed in Figure 3 A–C, the immunofluorescence assay using an antibody against 5-methylcytosine (5 mC) revealed that the bright 5 mC signals were along the entire 5S rDNA, indicating that the ∼1 Mb 5S rDNA arrays were hypermethylated. The 5 mC signals also extended to the 5S adjacent regions indicating that 5S rDNA located in a long range of high-level methylation region in *G. herbaceum*. This methylation pattern is similar with that observed in *Arabidopsis* [43] and rice [10], despite the centromeric part of rice 5S rDNA showing hypomethylated [10].

Telomere chromatin also exhibit epigenetic modifications including both histone and DNA methylation in vertebrates [44]. Unlike centromeric repeats, vertebrate telomeric TTAGGG repeat does not appear to be methylated due to the lack of canonical CpG sequence. In plants, cytosine in telomere is susceptible to methylation because cytosine in asymmetric CpNpNp motifs could be methylated [45]. As shown in Figure 3D, signals of 5 mC could be found on the telomeres of all three cotton species. However, some of telomeres showed relative weak signals in *G. herbaceum*, indicating a low level of cytosine methylation. Interestingly, the adjacent subtelomeric regions were also heavily methylated in *G. hirsutum*, whereas only few week dots were found in the subtelomeric regions in both *G. herbaceum* and *G. arboreum*. In both animals and plants, the high density of methylated CpG sites have been identified in the adjacent subtelomeric regions [44], [46], [47]. Therefore, our results might suggest that there was a heterochromatin-featured subtelomeric region on each chromosome of *G. hirsutum* but might not in *G. herbaceum* and *G. arboreum*.

In plant, efforts on telomere epigenetic were only conducted in few species [46], [47]. Recently researchers have suggested that the telomere chromatin of *Arabidopsis* had a euchromatin features with low level of 5 mC but not in tobacco [46], [47]. However, due to the existing of ITR, which has the same TTTAGGG sequence and is difficult to be distinguished from the telomeric TTTAGGG arrays, these results still be controversial [48], [49]. The ITRs were usually presumably located in the heterochromatin regions with heavily DNA methylation. If so, the 5 mC signals would be found on ITS and its both sides of adjacent regions, whereas the 5 mC signals will be found in only one adjacent region of telomere if it was methylated. As expected, our result proved it. In other words, fiber-FISH provide a more reliable method to analyze the DNA methylation on true telomere.

### FISH on BAC molecule

**Figure 4:**
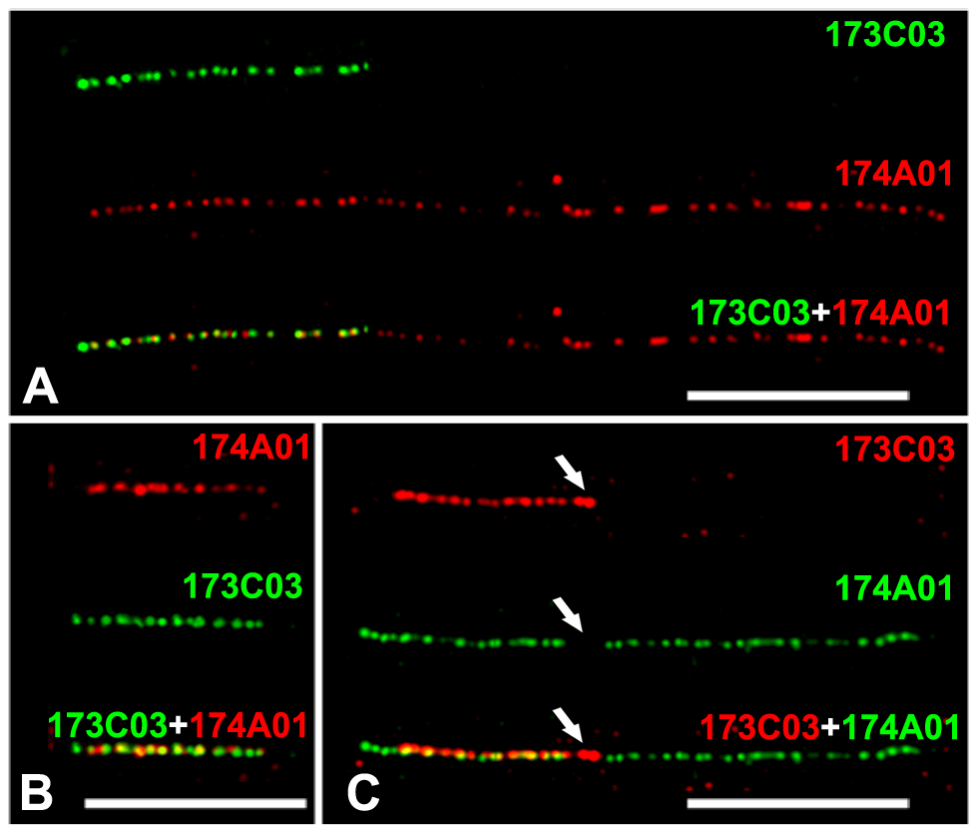
FISH on extended genomic DNA fibers and BAC molecules. A) FISH signals derived from two BAC clones 173C03 (green) and 174A01 (red) on extended genomic fibers of *G. hirsutum*. B) FISH mapping of BAC 173C03 molecules. Both clones 174A01 (red) and BAC 173C03 (green) were used as probe and hybridized onto 173C03 molecules to visualize the BAC 173C03 molecules and overlap regions. Red signals (174A01) covered most parts of BAC 173C03 molecules indicating that most parts of 173C03 were overlapped with 174A01. C) FISH mapping of BAC 174A01 molecules. Probes 174A01 (green) and BAC 173C03 (red) were hybridized onto 174A01 molecules. To facilitate the identification of overlapped regions, BAC vector (red, arrow) was labeled and hybridized simultaneously with two BAC probes. Bars are 10 µm.

The assembly of BAC/YAC clones or large contigs is the primary strategy for sequencing large complex genomes. Fiber-FISH is the easy and powerful way to assemble large fragments by constructing high-resolution physical map, especially for the fragments containing lots of repetitive sequences which are difficult to access by sequencing techniques [5], [50]. To test it, two BAC clones 173C03 and 174A01 which screened by the same molecular marker NAU1463 were used as probes in fiber-FISH. The size of two clones, 173C03 and 174A01, were measured as 41.7±6.6 µm and 14.8±3.5 µm (n = 12), corresponding to 135.0±21.3 kb and 47.9±11.2 kb, respectively. Interestingly, nearly the whole 173C03 (∼47.6 kb) was overlapped with 174A01 at the distal region (Figure 4A).

However, most of large insert clones are difficult to mapping on genome DNA fibers because they usually contain repetitive sequences, which occurred as high copy numbers in genome level. A clone molecule-based fiber-FISH has been developed to overcome this problem [3], [51]. However, when we tried it according to previously published protocols [3], [51], very few circular molecules were obtained (1–5 circular molecules per slide). In addition, the linear molecules vary greatly in size for the same BAC clone, suggesting that BAC molecules were broken into fragments of various sizes. To solve it, we developed a slight-stretching method here (see details in Method). BAC clones 68D15 and 259M16 were tested firstly. Both circular and linear molecules were obtained (Figure S1). The measurements from circular molecules showed a large variation (standard deviation, 13.2 µm for BAC 68D15 and 3.5 µm for 259M16, n = 20), whereas remarkable consistent results were obtained from the linear molecules (standard deviation, 2.5 µm for BAC 68D15 and 1.2 µm for 259M16) indicating that most of molecules were intact and were stretched uniformly (Table 1, Figure S1). Because linear molecules always attached on the glass in the direction of liquid flow, the stretching force could uniformly work on it in our drop coverslip extension method (see details in Methods) [51]. However, circular molecules formed various of geometrical shapes, and the stretching force could not be applied uniformly on each of side [52], which, therefore, caused large variation in total size estimations. So, only linear molecules were included in the further analysis. The average lengths (include 7.5-kb vector) of two BAC clones were calculated as 17.7 µm in BAC 68D15 and 7.7 µm in 259M16, determinating an average resolution as 5.74 kb/µm. This resolution is lower than that obtained from the genomic fiber (3.24 kb/µm) (Table 1), indicating relatively low level of stretching was applied on these BAC molecules. The slight stretching, therefore, kept most of linear molecules intact and not to be broken easily into pieces as other methods [3], [51], [52]. To further test it, we measured the molecule lengths of two BACs 173C03 and 174A01. The lengths of BAC 173C03 and 174A01 were revealed as 43.6±6.9 kb and 138.6±19.0 kb in molecule FISH, respectively, which were consistent with results obtained in genome fiber-FISH (*t*-test, *p*>0.19). As expected, when 174A01 was used as probe to hybridize onto 173C03 molecule, all the parts of molecule was stained (Figure 4B), whereas an overlapped region with 40.2±5.5 kb was detected when the 173C03 was hybridized onto 174A01 molecule (Figure 4C). Consistently, the overlapped region was closed to the vector, suggesting that it was on the distal region of 174A01 (Figure 4C). To further confirm it, HindIII fingerprinting was conducted. After laborious DNA digestion and delicate gel running, we were able to identify that most parts of clone 173C03 was overlapped with174A01 based on the similar band-pattern between two BACs (data not shown). However we could not identify where the overlapped region was, and how about the size of overlapped region unless the further sequencing was conducted. However, with the present technique we were able to visualize the BACs physical map, in which we can determine accurate size of both BACs and their relative locations.

## Materials and Methods

### Materials

A tetroploid cotton, *G. hirsutum* acc. TM-1 and two diploid species *G. arboreum* cv. JLZM and *G. herbaceum* race *kuljianum* cv. hongxincaomian were used for extended DNA preparation. All BACs were derived from the tetroploid cotton BAC libraries as described previously [20].

An *Arabidopsis thaliana* BAC F23H06 containing 42 copies of TTTAGGG was used to isolate *Arabidopsis* telomere by PCR. One pair of primer, Y7454, was designed using the online software Primer 3 (http://frodo.wi.mit.edu/) (Y7454F: 5′-AACGAGGTATCCCTACTATGA-3′; Y7454R: 5′-TGGTTCCCTTTCCACTA -3′). The PCR reaction was 94°C for 3 min, followed by 35 cycles of 95°C for 30 s, 58°C for 30 s, and 72°C for 45 s and ended by a 4 min extension at 72°C. PCR products were recovered by a Gel Extraction Kit (Qiagen catalog no. 28704).

### FISH on DNA fiber

Cotton has high endogenous levels of polysaccharides, phenolics, and other organic constituents that interfere with the nuclei isolation and further DNA fiber preparation. To solve this, a modified protocol was developed based on previous study [1]. Young plants were grown in greenhouse and keep in dark for two days before leaf collection. One gram young leaf was grinded into fine powder in liquid nitrogen. The powder was then incubated with 20 mL of cold nuclei isolation buffer (10 mM Tris-HCl (pH9.5), 10 mM EDTA, 100 mM KCl, 0.5 M Sucrose, 4.0 mM Spermidine, 1.0 mM Spermine, 0.1% mercaptoethanol, and 1% PVP40) on ice for 5 min. The solution was filtered immediately through 100, 50, and 30 µm mesh nylon membranes. After incubating with 750 µl 20% triton X-100 on ice for 5 min, the filtrate was centrifuged at 2000×g for 10 min at 4°C. The pellet was resuspended in 500-µl stock buffer (NIB : glycerol = 1∶1) and stored at −20°C. Extension of DNA fibers, hybridization and signal detection were performed as previous protocol [1].

### FISH on BAC clone molecule

BAC-DNA was isolated using the alkaline lysis method [53]. Approximately 10–20 ng BAC-DNA was diluted in 10 µl distilled water and then pipette onto a poly-L-lysine glass slide. To prevent molecule broken, a 18×18 coverslip was used to spread the molecules. One end of the coverslip was positioned on the slide firstly and the coverslip was gradually pressed down toward the slide until it covered the slide. The liquid flow between the slide and coverslip would extend the DNA. Slide was air-dried for 30 min at RT and then the coverslip was washed off in water. After air dry, slide was fix in 3∶1 100% ethanol: glacial acetic for 2 min and dried at 60°C for 30 min. Slides can be used immediately for FISH or stored in −20°C for several weeks. Probe hybridization and signal detection were same with above genomic fiber-FISH. To visualize the vector, about five- to 10-fold excess probe DNA was included in the hybridization mixture to out compete the homologous sequences in the total BAC probe.

### Combining fiber-FISH of 5S rNDA and immunodetection of 5 mC on extended DNA fibers

5S rNDA fiber-FISH combined with immunodetestion of 5 mC was conducted according to previously published protocol with a few modifications [10]. DNA fiber slides were firstly hybridized with biotin labeled 5S rDNA probe. In brief, FISH probe mixture (50% formamide, 10% dextran sulfate, 50 ng of labeled DNA in 2× SSC) was applied to the slide, covered with a coverslip, and sealed with rubber cement. The slide was then denatured for 3 min at 80°C and incubated at 37°C overnight. After hybridization the slide was washed in 2× SSC for 5 min at RT, 2× SSC for 5 min at 42°C, 2× SSC for 5 min at RT, and 1× PBS for 5 min at RT. To reduce background, slides were incubated with 0.5% BSA in 1×PBS for 30 min at 37°C. The slides were then incubated with the anti-5 mC antibody diluted 1∶100 in the blocking solution (0.5% BSA in 1×PBS) overnight at RT. Post-hybridization washing and signal detection was according to previous protocol [10].

### Cytological measurement and analysis

All images were captured digitally using an Evolution VF CCD camera (Media Cybernetics, USA) installed on an Olympus BX51 fluorescence microscope. Images were merged and measured using Image-Pro Express software V5.0 (Media Cybernetics, USA). Final image adjustments were performed using Adobe Photoshop 8. The path of the chromosomes was computationally traced and straightened according to the manual provided by the Image J software V1.41 (http://rsb.info.nih.gov/ij).

## Supporting Information

Table S1 5S rDNA length in *G. arboreum*.
(DOCX)

Figure S1 **Circular and linear molecules of BAC 259M16.** A) One microscopy field of BAC 259M16 contains different size of circle molecules. Several molecules with obvious large size variation are pointed out with arrows. The size of each molecule (in µm) was showed above the corresponding molecules. B) One microscopy field of BAC 259M16 contains five linear molecular with high consistency in size. The size of each molecule (in µm) was showed above the corresponding molecules. Bars are 10 µm.
(TIF)

## References

[1] Jackson SA, Wang ML, Goodman HM, Jiang J (1998) Application of fiber-FISH in physical mapping of *Arabidopsis thaliana* . Genome 41: 566–572. 9796106

[2] Fransz P, Alonso-Blanco C, Liharska TB, Peeters AJ, Zabel P, et al (1996) High-resolution physical mapping in *Arabidopsis thaliana* and tomato by fluorescence in situ hybridization to extended DNA fibres. Plant J 9: 421–430. 8919917 10.1046/j.1365-313x.1996.09030421.x

[3] Jackson SA, Dong F, Jiang J (1999) Digital mapping of bacterial artificial chromosomes by fluorescence in situ hybridization. Plant J 17: 581–587. 10205912 10.1046/j.1365-313x.1999.00398.x

[4] Cheng Z, Buell CR, Wing RA, Jiang J (2002) Resolution of fluorescence in-situ hybridization mapping on rice mitotic prometaphase chromosomes, meiotic pachytene chromosomes and extended DNA fibers. Chromosome Res 10: 379–387. 12296520 10.1023/a:1016849618707

[5] Jiang J, Gill BS (2006) Current status and the future of fluorescence in situ hybridization (FISH) in plant genome research. Genome 49: 1057–1068. 17110986 10.1139/g06-076

[6] Zhang H, Phan BH, Wang K, Artelt BJ, Jiang J, et al (2012) Stable integration of an engineered megabase repeat array into the maize genome. The Plant Journal 70: 357–365. 22233334 10.1111/j.1365-313X.2011.04867.x

[7] Gong Z, Wu Y, Koblizkova A, Torres GA, Wang K, et al (2012) Repeatless and Repeat-Based Centromeres in Potato: Implications for Centromere Evolution. Plant Cell 24: 3559–3574. 22968715 10.1105/tpc.112.100511 PMC3480287

[8] Cook DE, Lee TG, Guo X, Melito S, Wang K, et al (2012) Copy number variation of multiple genes at *Rhg1* mediates nematode resistance in soybean. Science 338: 1206–1209. 23065905 10.1126/science.1228746

[9] Schwarzacher T (2003) DNA, chromosomes, and in situ hybridization. Genome 46: 953–962. 14663512 10.1139/g03-119

[10] Koo DH, Han F, Birchler JA, Jiang J (2011) Distinct DNA methylation patterns associated with active and inactive centromeres of the maize B chromosome. Genome Res 21: 908–914. 21518739 10.1101/gr.116202.110 PMC3106323

[11] Kuznetsova I, Podgornaya O, Ferguson-Smith MA (2006) High-resolution organization of mouse centromeric and pericentromeric DNA. Cytogenetic and Genome Research 112: 248–255. 16484780 10.1159/000089878

[12] Blower MD, Sullivan BA, Karpen GH (2002) Conserved Organization of Centromeric Chromatin in Flies and Humans. Developmental Cell 2: 319–330. 11879637 10.1016/s1534-5807(02)00135-1 PMC3192492

[13] Wang K, Wang Z, Li F, Ye W, Wang J, et al (2012) The draft genome of a diploid cotton *Gossypium raimondii* . Nat Genet 44: 1098–1103. 22922876 10.1038/ng.2371

[14] Paterson AH, Wendel JF, Gundlach H, Guo H, Jenkins J, et al (2012) Repeated polyploidization of Gossypium genomes and the evolution of spinnable cotton fibres. Nature 492: 423–427. 23257886 10.1038/nature11798

[15] Wang K, Guan B, Guo W, Zhou B, Hu Y, et al (2008) Completely distinguishing individual A-genome chromosomes and their karyotyping analysis by multiple bacterial artificial chromosome-fluorescence in situ hybridization. Genetics 178: 1117–1122. 18287408 10.1534/genetics.107.083576 PMC2248359

[16] Wang K, Song X, Han Z, Guo W, Yu JZ, et al (2006) Complete assignment of the chromosomes of *Gossypium hirsutum* L. by translocation and fluorescence in situ hybridization mapping. Theor Appl Genet 113: 73–80. 16609860 10.1007/s00122-006-0273-7

[17] Hanson RE, Islam-Faridi MN, Percival EA, Crane CF, Ji Y, et al (1996) Distribution of 5S and 18S-28S rDNA loci in a tetraploid cotton (*Gossypium hirsutum* L.) and its putative diploid ancestors. Chromosoma 105: 55–61. 8662259 10.1007/BF02510039

[18] Ji Y, Zhao X, Paterson AH, Price HJ, Stelly DM (2007) Integrative mapping of *Gossypium hirsutum* L. by meiotic fluorescent in situ hybridization of a tandemly repetitive sequence (B77). Genetics 176: 115–123. 17409065 10.1534/genetics.107.071738 PMC1893065

[19] Zhao XP, Si Y, Hanson RE, Crane CF, Price HJ, et al (1998) Dispersed repetitive DNA has spread to new genomes since polyploid formation in cotton. Genome Res 8: 479–492. 9582192 10.1101/gr.8.5.479

[20] Wang K, Guo W, Yang Z, Hu Y, Zhang W, et al (2010) Structure and size variations between 12A and 12D homoeologous chromosomes based on high-resolution cytogenetic map in allotetraploid cotton. Chromosoma 119: 255–266. 20127105 10.1007/s00412-009-0254-0

[21] Wang K, Yang Z, Shu C, Hu J, Lin Q, et al (2009) Higher axial-resolution and sensitivity pachytene fluorescence in situ hybridization protocol in tetraploid cotton. Chromosome Research 17: 1041–1050. 19844799 10.1007/s10577-009-9085-3

[22] Heiskanen M, Hellsten E, Kallioniemi O-P, MÄKelÄ TP, Alitalo K, et al (1995) Visual Mapping by Fiber-FISH. Genomics 30: 31–36. 8595900 10.1006/geno.1995.0005

[23] Shiels C, Coutelle C, Huxley C (1997) Analysis of ribosomal and alphoid repetitive DNA by fiber-FISH. Cytogenet Cell Genet 76: 20–22. 9154117 10.1159/000134504

[24] Sjoberg A, Peelman LJ, Chowdhary BP (1997) Application of three different methods to analyse fibre-FISH results obtained using four lambda clones from the porcine MHC III region. Chromosome Res 5: 247–253. 9244452 10.1023/a:1018419619634

[25] Jin W, Melo JR, Nagaki K, Talbert PB, Henikoff S, et al (2004) Maize centromeres: organization and functional adaptation in the genetic background of oat. Plant Cell 16: 571–581. 14973167 10.1105/tpc.018937 PMC385273

[26] Endrizzi JE, Turcotte EL, Kohel RJ (1985) Genetics, Cytology, and Evolution of *Gossypium*. In: Caspari EW, John GS, editors. Advances in Genetics: Academic Press. pp. 271–375.

[27] Wendel JF, Schnabel A, Seelanan T (1995) An unusual ribosomal DNA sequence from *Gossypium gossypioides* reveals ancient, cryptic, intergenomic introgression. Molecular Phylogenetics and Evolution 4: 298–313. 8845966 10.1006/mpev.1995.1027

[28] Watson JM, Riha K (2010) Comparative biology of telomeres: Where plants stand. FEBS Letters 584: 3752–3759. 20580356 10.1016/j.febslet.2010.06.017 PMC3767043

[29] McKnight TD, Riha K, Shippen DE (2002) Telomeres, telomerase, and stability of the plant genome. Plant Mol Biol 48: 331–337. 11905960 10.1023/a:1014091032750

[30] Uchida W, Matsunaga S, Sugiyama R, Kawano S (2002) Interstitial telomere-like repeats in the Arabidopsis thaliana genome. Genes Genet Syst 77: 63–67. 12036106 10.1266/ggs.77.63

[31] O'Callaghan N, Dhillon V, Thomas P, Fenech M (2008) A quantitative real-time PCR method for absolute telomere length. Biotechniques 44: 807–809. 18476834 10.2144/000112761

[32] Saldanha SN, Andrews LG, Tollefsbol TO (2003) Assessment of telomere length and factors that contribute to its stability. European Journal of Biochemistry 270: 389–403. 12542689 10.1046/j.1432-1033.2003.03410.x

[33] Mizuno H, Wu J, Kanamori H, Fujisawa M, Namiki N, et al (2006) Sequencing and characterization of telomere and subtelomere regions on rice chromosomes 1S, 2S, 2L, 6L, 7S, 7L and 8S. Plant J 46: 206–217. 16623884 10.1111/j.1365-313X.2006.02684.x

[34] Di Giulio M (2010) A Model of the Origin of the 5S Ribosomal RNA Molecule. Journal of Molecular Evolution 71: 1–2. 20556372 10.1007/s00239-010-9358-7

[35] Sun F-J, Caetano-Anollés G (2009) The evolutionary history of the structure of 5S ribosomal RNA. Journal of Molecular Evolution 69: 430–443. 19639237 10.1007/s00239-009-9264-z

[36] Cronn RC, Zhao XP, Paterson AH, Wendel JF (1996) Polymorphism and concerted evolution in a tandemly repeated gene family: 5S ribosomal DNA in diploid and allopolyploid cottons. Journal of Molecular Evolution 42: 685–705. 8662014 10.1007/BF02338802

[37] Zhong XB, Fransz PF, Wennekes-Eden J, Ramanna MS, van Kammen A, et al (1998) FISH studies reveal the molecular and chromosomal organization of individual telomere domains in tomato. Plant J 13: 507–517. 9680996 10.1046/j.1365-313x.1998.00055.x

[38] Ohmido N, Kijima K, Ashikawa I, de Jong JH, Fukui K (2001) Visualization of the terminal structure of rice chromosomes 6 and 12 with multicolor FISH to chromosomes and extended DNA fibers. Plant Molecular Biology 47: 413–421. 11587512 10.1023/a:1011632111845

[39] Li E (2002) Chromatin modification and epigenetic reprogramming in mammalian development. Nat Rev Genet 3: 662–673. 12209141 10.1038/nrg887

[40] Cokus SJ, Feng S, Zhang X, Chen Z, Merriman B, et al (2008) Shotgun bisulphite sequencing of the Arabidopsis genome reveals DNA methylation patterning. Nature 452: 215–219. 18278030 10.1038/nature06745 PMC2377394

[41] Lister R, O'Malley RC, Tonti-Filippini J, Gregory BD, Berry CC, et al (2008) Highly integrated single-base resolution maps of the epigenome in Arabidopsis. Cell 133: 523–536. 18423832 10.1016/j.cell.2008.03.029 PMC2723732

[42] Zhong S, Fei Z, Chen YR, Zheng Y, Huang M, et al (2013) Single-base resolution methylomes of tomato fruit development reveal epigenome modifications associated with ripening. Nat Biotechnol 31: 154–159. 23354102 10.1038/nbt.2462

[43] Mathieu O, Yukawa Y, Sugiura M, Picard G, Tourmente S (2002) 5S rRNA genes expression is not inhibited by DNA methylation in Arabidopsis. The Plant Journal 29: 313–323. 11844108 10.1046/j.0960-7412.2001.01212.x

[44] Blasco MA (2007) The epigenetic regulation of mammalian telomeres. Nat Rev Genet 8: 299–309. 17363977 10.1038/nrg2047

[45] Meyer P, Niedenhof I, ten Lohuis M (1994) Evidence for cytosine methylation of non-symmetrical sequences in transgenic Petunia hybrida. EMBO J 13: 2084–2088. 8187761 10.1002/j.1460-2075.1994.tb06483.x PMC395059

[46] Majerova E, Fojtova M, Mozgova I, Bittova M, Fajkus J (2011) Hypomethylating drugs efficiently decrease cytosine methylation in telomeric DNA and activate telomerase without affecting telomere lengths in tobacco cells. Plant Mol Biol 77: 371–380. 21866390 10.1007/s11103-011-9816-7

[47] Vaquero-Sedas MI, Gamez-Arjona FM, Vega-Palas MA (2010) Arabidopsis thaliana telomeres exhibit euchromatic features. Nucleic Acids Research 39: 2007–2017. 21071395 10.1093/nar/gkq1119 PMC3064777

[48] Vaquero-Sedas MI, Vega-Palas MA (2011) DNA methylation at tobacco telomeric sequences. Plant Molecular Biology 77: 529–531. 22016003 10.1007/s11103-011-9833-6

[49] Majerová E, Fojtová M, Mandáková T, Fajkus J (2011) Methylation of plant telomeric DNA: what do the results say? Plant Molecular Biology 77: 533–536. 10.1007/s11103-011-9816-7 21866390

[50] De Jong JH, Fransz P, Zabel P (1999) High resolution FISH in plants - techniques and applications. Trends in Plant Science 4: 258–263. 10407441 10.1016/s1360-1385(99)01436-3

[51] Weier HU, Wang M, Mullikin JC, Zhu Y, Cheng JF, et al (1995) Quantitative DNA fiber mapping. Hum Mol Genet 4: 1903–1910. 8595414 10.1093/hmg/4.10.1903

[52] Henegariu O, Grober L, Haskins W, Bowers PN, State MW, et al (2001) Rapid DNA fiber techniques for size measurements of linear and circular DNA probes. BioTechniques 31: 246–250. 11515354 10.2144/01312bm01

[53] Sambrook J, Fritsch EF, Maniatis T (1989) Molecular cloning : a laboratory manual. Cold Spring Harbor, N.Y.: Cold Spring Harbor Laboratory.

