# Supplementary material for: Systematic Application of DNA Fiber-FISH Technique in Cotton
Source: PLoS One. 2013 Sep 27;8(9):e75674. doi: 10.1371/journal.pone.0075674 (PMC3785504; doi:10.1371/journal.pone.0075674)
Supplement: Table S1 — 5S rDNA length in G. arboreum. (DOCX) [file pone.0075674.s001.docx]

**Table S1. 5S rDNA length in *G.arboreum***

| **Number of fiber** | **Fiber size (µm)** | **Physical length (kb)** |
| --- | --- | --- |
| 1 | 900.8 | 2,918.4 |
| 2 | 923.6 | 2,992.5 |
| 3 | 925.2 | 2,997.7 |
| 4 | 951.3 | 3,082.3 |
| 5 | 1,035.6 | 3,355.4 |
| Average | 947.3±52.5 | 3,069.3±170.1 |
