# Supplementary figures and images for: Systematic Application of DNA Fiber-FISH Technique in Cotton
Source: PLoS One. 2013 Sep 27;8(9):e75674. doi: 10.1371/journal.pone.0075674 (PMC3785504; doi:10.1371/journal.pone.0075674)

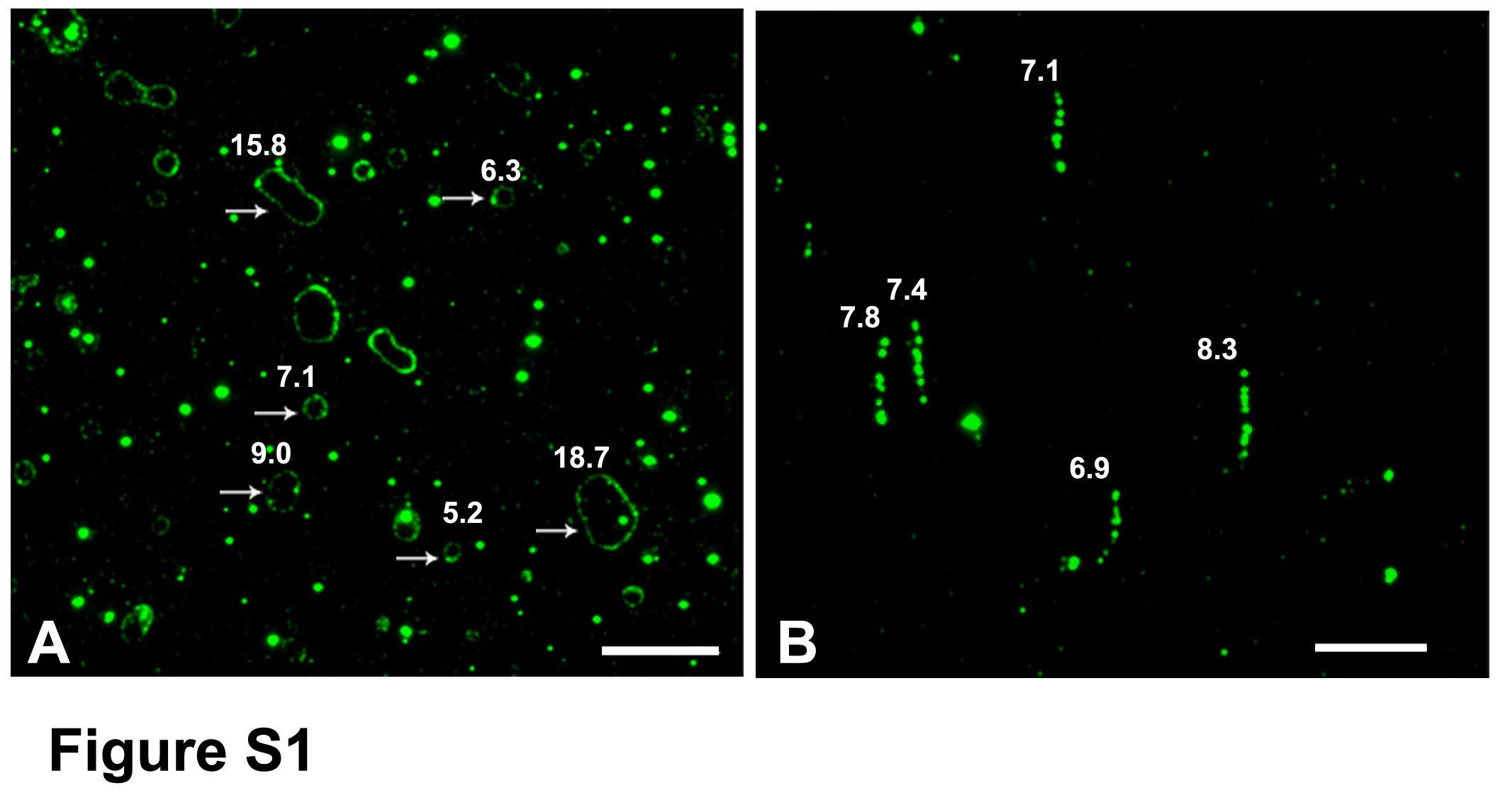

Supplement: Figure S1 — Circular and linear molecules of BAC 259M16. A) One microscopy field of BAC 259M16 contains different size of circle molecules. Several molecules with obvious large size variation are pointed out with arrows. The size of each molecule (in µm) was showed above the corresponding molecules. B) One microscopy field of BAC 259M16 contains five linear molecular with high consistency in size. The size of each molecule (in µm) was showed above the corresponding molecules. Bars are 10 µm. (TIF) [file pone.0075674.s002.tif]
